# Neural correlates of aesthetic tragedy: evidence for enhanced semantic processing and cognitive control in response to tragic versus joyful music

**DOI:** 10.3389/fpsyg.2025.1689581

**Published:** 2025-11-06

**Authors:** Chia-Wei Li, Chen-Gia Tsai

**Affiliations:** 1 Department of Radiology, Wan Fang Hospital, Taipei Medical University, Taipei, Taiwan; 2 Graduate Institute of Musicology, National Taiwan University, Taipei, Taiwan; 3 Graduate Institute of Brain and Mind Sciences, National Taiwan University, Taipei, Taiwan

**Keywords:** cognitive control, fMRI, musical emotion, semantic processing, tragedy

## Abstract

Philosophers and psychologists have long examined the paradox of negative emotions in art, yet its neural underpinnings remain insufficiently understood. This study employed functional magnetic resonance imaging to compare neural responses to the opening phrases of major- and minor-mode musical themes during the recapitulation section of first-movement sonata form. Compared to the major mode, the minor mode is generally associated with more negative emotional connotations. When presented with relatively greater loudness and a vigorous tempo, minor-mode themes often convey a sense of tragic force. Whole-brain analysis revealed significantly greater activation in the bilateral middle temporal gyrus (MTG), right lateral frontal pole (LFP), and right angular gyrus for tragic (minor-mode) compared to joyful (major-mode) themes. Elevated MTG activation suggests increased semantic processing demands involved in the construction of socio-affective meaning in response to tragic themes, whereas enhanced LFP activation likely reflects the engagement of high-level cognitive control and emotion regulation mechanisms. Complementary qualitative analyses of participants’ written responses further indicated that tragic themes more frequently elicited imagery of external oppression and internal emotional turmoil or uncertainty—patterns not observed for joyful themes. These findings provide neuroscientific support for the Distancing–Embracing model of the enjoyment of negative emotions in art reception.

## 1 Introduction

In everyday life, people typically avoid situations that evoke negative emotions. Yet, in the context of art, people often find aesthetic value in experiencing these very emotions. This seeming contradiction is known as the “paradox of negative emotion in art,” a phenomenon that has been extensively explored by philosophers (Carroll, 1990; Curran, 2001; Smuts, 2009; Levinson, 2011). Recent psychological models offer explanations for this paradox. The Distancing-Embracing model identifies two cognitive mechanisms underlying the appreciation of artworks that evoke negative emotions: (1) maintaining psychological distance from the negative affect and (2) embracing and deepening the experiential richness of these emotions through integrative meaning-making (Menninghaus et al., 2017). Consistent with this model, Ozbay et al. (2025) found that when participants anticipated strong negative emotions from certain content, they tended to avoid non-artistic presentations but were more inclined to engage with artistic depictions of the same material. The study suggested that participants expected artistic depictions to offer greater cognitive insights, which in turn motivated them to approach these works despite their negative content. These findings imply that the value of negatively valenced content may stem primarily from the meaning-making processes it elicits in the audience.

Instrumental music offers a unique avenue for exploring negative emotions in aesthetic contexts. Unlike vocal music, instrumental music often lacks explicit semantic content, yet it powerfully evokes a range of affective responses. Deeply rooted in historical and aesthetic traditions, the tragic affect in European classical music conveys drama, urgency, suffering, and sublimity. These elements reflect life’s hardships, conflicts, and experiences of oppression, sometimes leading to emotional catharsis or a sense of spiritual transcendence (Brook, 1970; Fenby-Hulse, 2014; Steinberg, 2014). Importantly, “tragic” is not synonymous with “sad.” While sadness in music, as well as in vocal expressions such as lamenting, sighing, or weeping, is often conveyed through soft dynamics and slow tempi (Juslin and Timmers, 2010), tragic music tends to be louder, bass-heavier, faster, and more rhythmically driven.

The tragic affect is particularly pronounced in the minor-mode first-movement sonata form. Major and minor modes are generally associated with positive and negative emotional connotations, respectively. This distinction is partly attributed to the heightened dissonance and tonal instability characteristic of minor-mode music compared to its major-mode counterpart (Parncutt, 2014). Additionally, the vigorous tempo typical of the first-movement sonata form further amplifies its emotional intensity. Consequently, when set in a minor key and presented with sufficiently high loudness, the movement’s primary theme often acquires a tragic quality.

Structurally, the sonata form comprises three principal sections: the exposition, development, and recapitulation. The exposition presents thematic material, the development heightens musical tension through harmonic and motivic transformation, and the recapitulation reinstates the primary and secondary themes, creating a sense of resolution (Rosen, 1971). However, in minor-mode pieces, this resolution does not offer emotional relief but instead clarifies the tragic inevitability—the tragic force is no longer in flux but fully realized (Hepokoski and Darcy, 2006). The return of the primary theme reaffirms the inevitability of suffering and the dominance of fate. For instance, in the first movement of Beethoven’s Symphony No. 5 in C minor, the conclusion of the development section evokes the image of a protagonist relentlessly pursued by fate, desperately seeking refuge as ominous knocking resounds. The ensuing recapitulation heightens the sense of fatal inevitability, as the force metaphorically bursts through the door with overwhelming certainty. This moment likely engages listeners’ cognitive control mechanisms to regulate the emotional weight of this realization, while semantic processing aids in framing the tragic inevitability within a socio-affective context. This is consistent with the Distancing-Embracing model, which suggests that negative emotions in art can facilitate emotional reappraisal and meaning-making (Menninghaus et al., 2017).

The Distancing-Embracing model posits that in tragic art, negative emotions are not merely sources of suffering but also serve as symbols of human dignity and existential significance. This raises a critical question: does meaning-making in response to tragedy involve an immediate cognitive transformation that renders negative events comprehensible and aesthetically appreciable—such as the recapitulation of the primary theme in the first movement of Beethoven’s Symphony No. 5—or does it entail a longer-term attribution of profound and enduring significance, as in the ultimate sense of triumph one may feel after experiencing the entire symphony? The Distancing-Embracing model appears to acknowledge this distinction, suggesting that meaning can be derived either in the moment or retrospectively, with both pathways enabling a positive reappraisal of negative events (Menninghaus et al., 2017). However, the model does not elaborate on the specific differences between these two processes, which may correspond to immediate versus reflective responses. Distinguishing immediate from reflective responses is especially important for tragic music. Whereas sad music tends to elicit reflective engagement—evoking negative affect “at a distance” and allowing grief exploration without real-life consequences (Taruffi and Koelsch, 2014)—tragic themes convey urgency and plausibly yield a more salient immediate response, potentially amplifying early meaning construction and regulatory engagement.

The present study explored how the brain rapidly constructs meaning and regulates affect at the onset of tragic musical passages. Using fMRI, we measured neural responses to the opening of tragic (minor-mode) and joyful (major-mode) themes presented at the start of the recapitulation in first-movement sonata form. We reasoned that tragic themes would elicit stronger negative affect and thus be accompanied by greater cognitive engagement than joyful themes. Such engagement should involve increased recruitment of systems supporting rapid semantic integration and high-level control, which jointly scaffold the initial construction of socio-affective meaning and the down-regulation of negative responses. By contrast, joyful themes—being more affectively consonant and semantically less demanding—were expected to place lower demands on these systems.

This expected contrast led to two specific predictions, which we tested using a whole-brain analytical approach. First, we predicted greater activity in the middle temporal gyrus (MTG) for tragic than for joyful themes, indexing heightened demands for meaning construction. These regions have been implicated in semantic processing and in integrating conceptual and episodic information into socio-affective meaning (Straube et al., 2012; van Mulukom et al., 2013; Dwyer et al., 2014; Jastorff et al., 2015; St Jacques et al., 2018; Jackson, 2021). Second, we predicted greater activity in frontal control regions for tragic than for joyful themes, with a specific focus on the right lateral frontal pole (LFP). This region is a key candidate node, given its established role in reappraisal-based regulation of negative affect (Krendl et al., 2012; Bramson et al., 2020) and modulation of pain perception (Petrovic et al., 2010; Peng et al., 2018). As tragic music can engage processes akin to vicarious psychological pain, we expected stronger LFP responses to tragic than to joyful themes.

To gain a more nuanced understanding of the subjective experience of aesthetic tragedy, we complemented the fMRI data with a qualitative analysis of participants’ self-generated descriptions. Several weeks before the fMRI session, participants listened to each excerpt and provided written descriptions of musical features, personal impressions, imagery, and emotions evoked by the music. These descriptions were then subjected to a thematic analysis, focusing on two key dimensions derived from the core characteristics of tragedy: (1) external oppression or challenge and (2) internal emotional turmoil or uncertainty. This framework allows us to explore the correspondence between the interpretation of “external oppression” and semantic processing, and between the management of “internal emotional turmoil” and cognitive control. This integrative approach, combining neuroimaging data with qualitative insights, offers a more comprehensive understanding of the neural and experiential foundations of aesthetic responses to musical tragedy.

## 2 Methods

This study presents a reanalysis of data from a previous experiment; additional methodological details—including participant recruitment, inclusion/exclusion criteria, and stimulus selection procedures—are described in our 2022 article (Li and Tsai, 2022).

### 2.1 Participants

Participants were recruited online and were required to have extensive familiarity with Western classical music. An online questionnaire was administered to assess participants’ emotional and cognitive responses to eight musical excerpts from sonata-form compositions, which served as the stimuli for the present study. Participants were presented with musical excerpts in alternating minor and major modes. Following each excerpt, they were instructed to first document their subjective impressions of the final passage of the development section and the recapitulation of the primary theme. Subsequently, they were tasked with interpreting these impressions by linking them to their objective musical sources, such as tempo, tonality, melody, rhythm, timbre, and dynamics. This process of articulating listening impressions and musico-affective attribution often yielded responses incorporating spontaneous scene imagery and detailed emotional state descriptions. In the present study, these feature–impression mappings were used solely for participant screening and were not subjected to formal quantitative analysis.

Based on the questionnaire results, participants were screened for eligibility to participate in the neuroimaging experiment by the corresponding author, who is a trained musicologist. Participants were selected based on three criteria: (1) they experienced strong anticipation in at least five of the eight transitional passages; (2) they perceived a strong sense of resolution in at least five of the eight recapitulations; and (3) they provided musically relevant explanations for at least five excerpts. Participants meeting all three criteria qualified for the neuroimaging experiment.

Initially, 31 adult volunteers completed the questionnaire for this study, and 27 of them met the screening criteria. Among these, 20 participants advanced to the fMRI experiment. All participants were free of neurological, psychiatric, or auditory disorders, and no structural abnormalities were observed in their brains. Written informed consent was obtained from each participant before the study, and they were compensated approximately 16 USD after the fMRI scan. Data from two participants were excluded due to excessive head movement during the scans. Thus, the final sample analysed in this study consisted of 18 participants (12 females aged 27.0 ± 6.7 years, range 20–40 years) with an average of 10.28 years (SD = 4.78) of musical instrument training.

### 2.2 Stimuli

The musical stimuli consisted of four minor-mode and four major-mode excerpts selected from the first movements of Western classical masterworks. These excerpts were chosen based on several criteria: the compositions adhered to sonata form, had tempi exceeding 90 beats per minute (BPM), and included a development-section closing passage showing a resolution-seeking surge immediately prior to the recapitulation of the primary theme. Additionally, the primary themes themselves were required to demonstrate moderate to high intensity, with a clear distinction between tragic (minor-mode) and joyful (major-mode) thematic characteristics. The minor-mode excerpts were selected from Joseph Haydn’s Symphony No. 45 (‘Farewell’) in F-sharp minor, Hob. I:45, Wolfgang Amadeus Mozart’s Symphony No. 25 in G minor, K. 183/173dB, Ludwig van Beethoven’s Symphony No. 5 (‘Fate’) in C minor, Op. 67, and Johannes Brahms’s Piano Concerto No. 1 in D minor, Op. 15. The major-mode excerpts were drawn from Mozart’s Concerto for Flute, Harp, and Orchestra in C major, K. 299/297c, Felix Mendelssohn’s Symphony No. 4 (‘Italian’) in A major, Op. 90, Beethoven’s Symphony No. 7 in A major, Op. 92, and Beethoven’s Piano Concerto No. 5 (‘Emperor’) in E-flat major, Op. 73.

Each selected excerpt lasted 30 s and focused on the recapitulation of the primary theme within a first-movement sonata form. The excerpts were sourced from commercially available recordings and digitized at a sampling rate of 44,100 Hz with 16-bit stereo quality using GoldWave (version 5.58, GoldWave Inc., Newfoundland, Canada). Each excerpt included an 18-s segment drawn from the final bars of the development section transitioning into the recapitulation of the primary theme, followed by a 12-s segment featuring the recapitulated theme itself.

To prevent participants from experiencing sustained exposure to emotionally intense musical stimuli, we also created atonal sequences using Reason software (version 8.0, Propellerhead Software Inc., Sweden). These atonal sequences matched the musical excerpts in duration (30 s) and were similarly digitized at 44,100 Hz and 16-bit stereo quality. All stimuli—both musical excerpts and atonal sequences—were normalized for loudness and modified with a 1-s fade-in and a 1-s fade-out to minimize abrupt auditory transitions.

### 2.3 Procedure

Prior to the MRI scan, participants listened to all musical stimuli and completed a questionnaire. They were asked to rate their agreement with four statements on a 7-point Likert scale (1 = entirely disagree, 2 = mostly disagree, 3 = somewhat disagree, 4 = neither agree nor disagree, 5 = somewhat agree, 6 = mostly agree, 7 = entirely agree) after the presentation of each stimulus: (1) I am very familiar with this music; (2) I felt strong anticipation before the theme came out; (3) The theme is very tragic sounding; and (4) The theme is very joyful sounding.

Participants wore earplugs to reduce scanner noise and scanner-compatible headphones to receive the auditory stimuli. Before the first scanning session, a 5-s excerpt from Mozart’s Clarinet Concerto was used to adjust the playback volume to a comfortable level for each participant, based on their feedback. This also allowed participants to acclimatize to the scanner noise and practice listening to music in the MRI environment.

The study comprised five fMRI scanning sessions, with 1-min rest intervals between them. Three sessions featured musical stimuli, alternating with two sessions from an unrelated linguistic study. This alternating design minimized affective habituation from repeated exposure to emotional music. During each musical session (approximately 7 min), participants listened to four minor-mode, four major-mode, and four atonal excerpts, presented in a pseudo-randomized order. Participants were instructed to keep their eyes closed throughout to maximize focus and immersion. No additional tasks or questions were given during these sessions to ensure undivided attention to the music.

### 2.4 MRI data acquisition

The experiments were performed using a Siemens 3 T MRI PRISMA at National Taiwan University. A gradient-echo echo-planar imaging (EPI) sequence was used in the functional data scanning. Approximately 2.5-mm slices of axial images were acquired in the functional scans using gradient echo planar imaging with the following parameters: time to repetition = 2,500 ms, echo time = 30 ms, flip angle = 87°, in-plane field of view = 192 × 192 mm, and acquisition matrix = 78 × 78 × 45, to cover all cerebral areas. Magnetization-Prepared Rapid Gradient Echo T1-weighted imaging with an isotropic spatial resolution of 0.9 mm was acquired in each participant for spatial individual-to-template normalization.

### 2.5 Data extraction, analysis, and synthesis

Normality of all rating variables—familiarity, anticipation, and perceived tragic and joyful qualities (for minor and major themes)—was assessed with the Shapiro–Wilk test; several variables deviated from normality (*p* < 0.05). We therefore conducted Wilcoxon signed-rank tests to perform two main analyses. First, we assessed whether scores for six dimensions—familiarity, anticipation, perceived tragic and joyful qualities for minor-mode themes, and perceived tragic and joyful qualities for major-mode themes—differed significantly from the neutral midpoint of 4. Second, we tested for a significant difference between familiarity ratings for minor- versus major-mode excerpts. To account for the seven comparisons, a Bonferroni correction was applied, setting the significance threshold at *p* < 0.0071 (0.05/7).

Participants’ descriptions of their personal impressions, imagery, and emotions evoked by the music were analyzed using a qualitative thematic approach to gain deeper insights into their subjective experiences of aesthetic tragedy. Given the study’s focus on the recapitulation of the primary theme, only participants’ descriptions of this section and their interpretations based on musical features were included in the analysis. Subjective impressions of the final passage of the development section, along with their corresponding musical interpretations, were excluded.

To prepare the data for analysis, the corresponding author segmented each participant’s questionnaire responses into smaller units when appropriate. Descriptions containing multiple contextual or logical elements were divided into separate entries, while maintaining the semantic coherence of each unit. After segmentation, a total of 58 descriptions were identified for tragic themes and 59 for joyful themes, with word counts ranging from 5 to 94 (including punctuation). This variation in length was largely attributable to the absence of a minimum word requirement, which allowed participants to provide highly concise responses.

The subsequent coding process was conducted by the corresponding author and a research assistant. The research assistant was blinded to the experimental stimuli to minimize expectancy and confirmation biases and to ensure that decisions were based solely on the textual content of each description. Both the corresponding author and the research assistant adhered strictly to predefined coding criteria. Each description was evaluated using binary coding, where ‘1’ indicated that the criterion was met and ‘0’ indicated that it was not met. Presence/absence coding is a standard operationalization in thematic/content analysis when categories are conceptually specified *a priori* and units vary in length, facilitating reliable aggregation and contingency testing (Manganello et al., 2010; Vaismoradi et al., 2013). The coding criteria were as follows:

(1) External Oppression or Challenge: This category encompassed descriptions of oppressive or threatening external forces or environments (e.g., fate, villains, tragic events, storms) impacting an individual, or the inherently threatening nature of such forces/environments even without a specified individual. Interpersonal conflict (e.g., arguments, blame, confrontations) was also included, representing external challenges causing psychological pressure. The music itself, if described as oppressive (e.g., evoking breathlessness), was also coded as ‘1’. Descriptions merely highlighting grandeur, magnificence, or overwhelming presence, without an oppressive impact, were coded as ‘0’.
(2) Internal Emotional Turmoil or Uncertainty: A description was coded as ‘1’ if it reflected a state of emotional instability or inner conflict. These included sudden emotional reactions (e.g., shock, surprise, astonishment), anxiety-related states (e.g., tension, unease, fear), and deep psychological distress (e.g., inner struggles, depression, doubt). Descriptions reflecting confusion, doubt, or the disruption of positive mood or pleasant circumstances, were also included.

All descriptions were shown in a pseudo-random order to minimize sequence effects. We pooled all entries and permuted them using a computer-generated sequence, presenting the same order to both raters. To ensure rigorous and unbiased evaluation, each description was coded independently. Prior to the independent coding process, the two raters were allowed to discuss the coding criteria and clarify category definitions to ensure consistency in interpretation.

To assess the reliability of thematic coding, we calculated percent agreement between two independent raters who evaluated all participant descriptions using binary labels. Following the reliability assessment, Fisher’s Exact Test was conducted to examine whether the presence of each coded theme (based on rater consensus) was significantly associated with the type of musical stimulus.

### 2.6 Analysis of FMRI data

Preprocessing and statistical analyses of the fMRI data were conducted using SPM12 (Wellcome Trust Centre for Neuroimaging; http://www.fil.ion.ucl.ac.uk/spm) and the Artifact Detection Tools (ART) toolbox. To mitigate magnetic saturation effects, the first four volumes of each scan were discarded. The remaining images were corrected for head motion and slice timing, co-registered to the participant’s anatomical scan, normalized to the MNI template, and resampled to 2-mm isotropic voxels. Spatial smoothing was performed using a 5-mm full-width at half-maximum (FWHM) Gaussian kernel. The ART toolbox was used to identify outlier volumes based on global intensity and motion parameters. A total of 18 participants whose head motion was below 2.5 mm (translation) and 1° (rotation), and whose scans were free of significant outliers, were included in the final analysis.

A whole-brain mass-univariate analysis was conducted within a two-level random-effects framework. At the first level, a general linear model (GLM) was fitted to each participant’s time series, modeling neural responses with the canonical hemodynamic response function (HRF). Events for all three conditions—minor-mode (Tragic), major-mode (Joyful), and the atonal music (Atonal)—were modeled identically: onsets were time-locked to 2 s after the beginning of the target segment (i.e., 25 s from excerpt onset), corresponding to the recognition window for the recapitulated theme in tonal excerpts and the matched time point in the atonal excerpts. This event was modeled as having zero duration. Six head motion parameters (three translations and three rotations) were included as nuisance covariates. To reduce potential confounding from acoustic and musical-structural features and isolate mode-specific effects, we entered RMS loudness, tempo (BPM), spectral centroid, roughness, and tonal stability as *z*-scored parametric modulators on the event regressor. These features were averaged over a 10-s window (−3 to +7 s relative to recapitulation onset) that brackets the modeled event and its immediate pre−/post-context. Feature extraction for RMS loudness, spectral centroid, and roughness was performed with MIRtoolbox. Tempo and tonal stability—operationalized as the proportion of time in chromatic harmony within the 10-s window—were determined by the corresponding author, a musicologist. Temporal autocorrelation was addressed using SPM’s fast autoregressive (FAST) model for pre-whitening procedure.

At the group level, whole-brain inference used paired *t*-tests comparing the three experimental conditions. Maps were thresholded at voxelwise *p* < 0.001 (uncorrected) and at a cluster-level AlphaSim-corrected threshold of *p* < 0.05 to control for multiple comparisons.

## 3 Results

### 3.1 Behavioral results

The Wilcoxon signed-rank tests showed that ratings were significantly above the neutral midpoint of 4 for familiarity (5.61 ± 1.19; *p* = 6.50 × 10^−22^), anticipation (5.72 ± 1.05; *p* = 3.12 × 10^−27^), the joyful quality of major-mode themes (5.97 ± 0.98; *p* = 1.30 × 10^−14^), and the tragic quality of minor-mode themes (5.53 ± 1.44; *p* = 4.83 × 10^−14^). In contrast, ratings were significantly below 4 for the tragic quality of major-mode themes (2.83 ± 1.83; *p* = 3.60 × 10^−7^) and the joyful quality of minor-mode themes (1.62 ± 0.78; *p* = 2.35 × 10^−14^). Together, these results indicate that participants were familiar with the excerpts, experienced strong anticipation before recapitulation, and reliably associated major/minor modes with joyful/tragic affect, respectively.

Inter-rater reliability was assessed using percent agreement. The agreement rate was 98.29% for the “external oppression or challenge” category and 100% for the “internal emotional turmoil or uncertainty” category, indicating high consistency between raters. These results suggest that the thematic coding was both reliable and robust.

To evaluate whether the presence of these semantic themes was associated with the emotional character of the musical stimuli (tragic vs. joyful), Fisher’s Exact Tests were conducted based on rater consensus. For “external oppression or challenge,” 28 out of 58 tragic descriptions met the consensus criterion, compared to 0 out of 59 joyful descriptions, yielding a highly significant association (*p* = 3.68 × 10^−11^). For “internal emotional turmoil or uncertainty,” 36 out of 58 tragic descriptions reached consensus, whereas only 1 out of 59 joyful descriptions did so, also yielding a strong statistical association (*p* = 8.54 × 10^−14^). These results support the hypothesis that tragic themes are more likely to elicit listener interpretations involving both oppressive external forces and internal psychological conflict, thereby validating the conceptual relevance of the thematic categories used.

### 3.2 FMRI data

**Figure 1:**
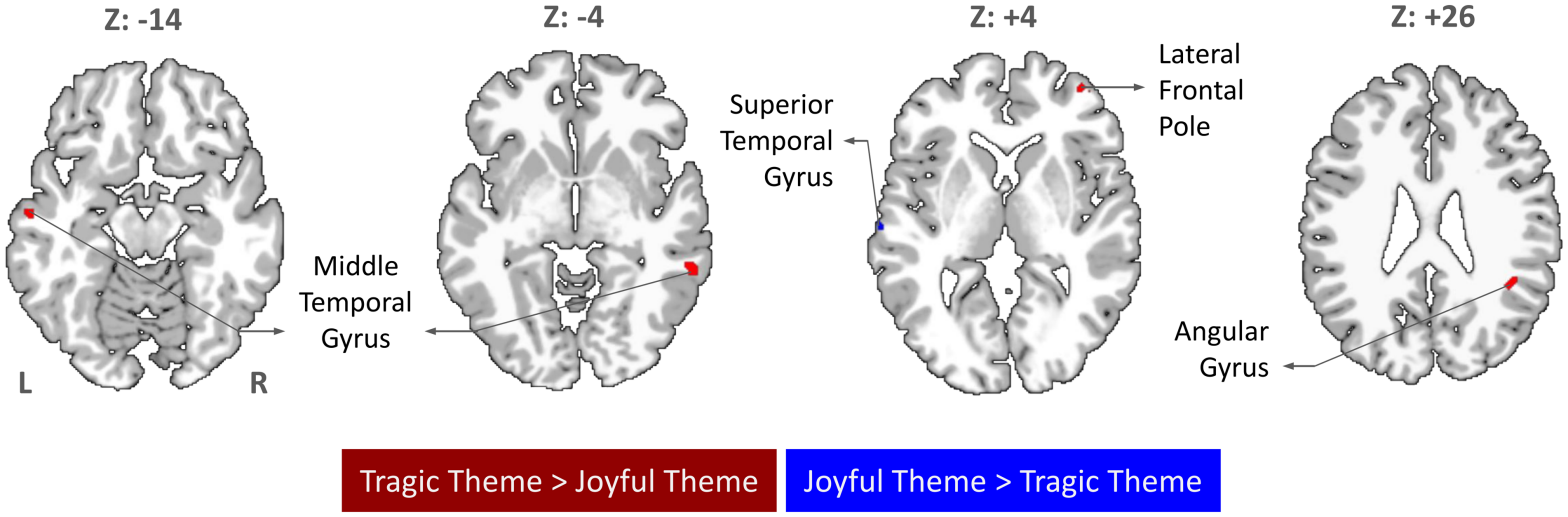
Whole-brain activation for tragic (minor-mode) versus joyful (major-mode) themes (voxelwise *p* < 0.001, uncorrected; cluster-level AlphaSim-corrected *p* < 0.05).

Using whole-brain random-effects analysis, including RMS loudness, tempo, spectral centroid, roughness, and tonal stability as covariates yielded no suprathreshold clusters attributable to these features in either Tragic or Joyful. The Tragic > Joyful contrast showed greater activity in the bilateral MTG, the right angular gyrus, and the right LFP (Figure 1; Table 1). Conversely, the Joyful > Tragic contrast revealed greater activation in the left superior temporal gyrus. The Joyful > Atonal contrast yielded greater activity in the superior temporal gyrus, whereas the Tragic > Atonal contrast revealed clusters in cerebellar lobules III and VI, the anterior cingulate cortex, and the superior temporal pole (Table 1). No suprathreshold clusters were observed for Atonal > Tragic or Atonal > Joyful.

**Table 1:** Whole-brain contrasts for listening to tragic (minor-mode) themes, joyful (major-mode) themes, and atonal music (voxelwise *p* < 0.001, uncorrected; cluster-level AlphaSim-corrected *p* < 0.05).

Volume information	Peak location (MNI)			T-value	Cluster size (voxel)
	X	Y	Z		
Tragic > Joyful					
Middle temporal gyrus	−58	−12	−16	7.40	23
	62	−42	−4	5.77	24
Middle frontal gyrus (lateral frontal pole)	46	48	4	4.77	21
Angular gyrus	44	−46	28	4.72	20
Joyful > Tragic					
Superior temporal gyrus	−60	−6	0	8.20	53
Tragic > Atonal					
Cerebellar lobule VI	24	−56	−26	6.73	57
Cerebellar vermis III	0	−40	−8	5.38	24
Anterior cingulate cortex	−4	16	26	4.88	21
Superior temporal pole	−52	16	−6	4.58	29
Joyful > Atonal					
Precentral gyrus	54	−2	50	4.72	25

## 4 Discussion

Since Aristotle’s *Poetics*, tragedy has been central to Western aesthetics, yet its neural underpinnings remain poorly understood. The present study used fMRI to investigate brain responses to the recapitulation of tragic and joyful themes in first-movement sonata form, with the goal of identifying the neural correlates of tragic affect. As predicted, tragic themes elicited significantly greater activation in the bilateral MTG and right LFP compared to joyful themes. Complementary qualitative analyses further revealed that tragic themes prompted listeners to construct interpretive narratives involving external oppression or challenge and internal emotional turmoil or uncertainty, whereas joyful themes did not elicit such complex affective scenarios. Together, these findings suggest that the bilateral MTG and right LFP are key regions supporting meaning-making and cognitive regulation when listeners are confronted with emotionally intense, aesthetically tragic musical material.

### 4.1 Activity in bilateral MTG

Our finding of greater activation in the middle portion of the left MTG during the theme recapitulation of tragic compared to joyful music is consistent with contemporary accounts of a left-lateralized semantic control network, in which posterior lateral temporal cortex constitutes a core node (Jackson, 2021). The middle left MTG supports the access to multimodal conceptual knowledge and the transformation of sensory input into interpretable semantic representations (Dwyer et al., 2014; Jastorff et al., 2015). In the context of tragic music, the recapitulation of a familiar theme may trigger emotionally charged semantic associations, activating stored knowledge related to oppressive external forces or internal emotional turmoil and uncertainty. For example, in the present study, the recapitulation of tragic themes in Mozart’s Symphony No. 25 and Haydn’s Symphony No. 45 featured a sharp increase in rhythmic momentum, embodying the musical style of *Sturm und Drang*. Participants described these passages with vivid expressions such as “a stampede of horses” and “an urgent, anxious tension,” reflecting the emotional intensity and dynamic unrest characteristic of this stylistic tradition.

Beyond its role in general semantic processing, the middle left MTG has also been implicated in the use of semantic information and retrieved episodic details for the construction of coherent mental representations. Schacter and Addis (2007) proposed the Constructive Episodic Memory Hypothesis, which posits that the human memory system is inherently flexible, allowing past episodic details to be recombined in novel ways to support both retrospective recollection and prospective simulation. Supporting this view, Addis et al. (2009) reported robust left MTG activation during both the imagination of future and past events, suggesting its crucial role in the recombination of episodic elements drawn from memory stores. Similarly, van Mulukom et al. (2013) found that the MTG contributes to future event simulation by integrating semantic knowledge with episodic details to construct plausible scenarios. Notably, St Jacques et al. (2018) demonstrated that the left MTG is involved not only in recalling autobiographical memories but also in constructing counterfactual versions of past events, highlighting its function in memory reconfiguration and context-based reinterpretation. In the present study, the recapitulation of tragic themes may have similarly prompted listeners to reinterpret musical material by retrieving and reorganizing relevant episodic details. For example, participants described the tragic theme of the first movement of Brahms’s Piano Concerto No. 1 with vivid imagery such as “a dark cavern” and “a torrential downpour with thunder in a desolate wilderness.” These responses suggest that listeners engaged both episodic and semantic memory systems to construct emotionally charged mental simulations—scenarios rooted in everyday experiences yet imbued with a sense of the fantastical.

Given that participants in our study generated a comparable number of interpretive narratives for both tragic and joyful musical themes, the stronger activation of the middle left MTG in response to tragic themes is unlikely to result merely from differences in the quantity of narrative elaboration. Rather, this likely reflects the distinct emotional valence and greater contextual and self-referential demands elicited by tragic content. Prior research has demonstrated that the middle left MTG is recruited during morally complex evaluations, such as when individuals make judgments about personal moral transgressions (Greene et al., 2004). In these contexts, the middle left MTG is thought to facilitate contextual semantic processing by integrating emotionally salient and socially meaningful information. Similarly, Yoshimura et al. (2009) reported greater bilateral MTG activation during self-referential evaluations of negative trait adjectives, with the middle left MTG specifically implicated in retrieving semantic memory relevant to the self and integrating affect-laden concepts and experiences. These findings suggest that the middle left MTG plays a critical role in constructing semantically coherent interpretations of personally relevant, emotionally negative stimuli. In the present study, tragic music may have evoked such self-relevant semantic processing, prompting listeners to draw upon internal representations of emotional turmoil and uncertainty. In contrast, joyful music—while eliciting interpretive engagement—was less likely to involve the same degree of affective complexity or self-referential processing.

In the context of negative emotion processing, the left MTG likely contributes to emotion regulation, particularly through its involvement in semantic retrieval, conceptual organization, and cognitive reappraisal. The left MTG has been shown to interact with prefrontal regions responsible for cognitive control. During free recall tasks, it operates in conjunction with the dorsolateral (DLPFC) and ventrolateral prefrontal cortex (VLPFC) to support semantic retrieval and the strategic organization of episodic content (Long et al., 2010). Consistently, Otto et al. (2014) identified the left MTG as a region commonly activated during both cognitive reappraisal and top-down generation of emotional states, underscoring its role in the reinterpretation and construction of affectively salient meaning. Within a broader regulatory network, the left MTG was found to exhibit functional coactivation with the DLPFC and VLPFC—regions known for implementing cognitive control. This mechanism is presumed to be engaged not only during the regulation of emotional events in the real world but also during the processing of symbolically rich artistic stimuli, such as tragic music. In this context, the left MTG may operate in concert with prefrontal control regions to facilitate the interpretive elaboration of musical meaning.

Beyond the left MTG, the right posterior MTG also showed greater activation for tragic than for joyful themes, albeit with a smaller effect size. An fMRI study of speech-and-gesture semantics reported a left-lateralized supramodal semantic network, alongside a smaller right posterior MTG cluster present for speech semantics and in the speech–gesture overlap, suggesting a stable but small-scale contribution (Straube et al., 2012). Convergent structural and resting-state connectivity from a tractography-based parcellation places the right posterior MTG in networks linking perisylvian language regions, compatible with a role in semantic processing (Xu et al., 2015). Notably, the right posterior MTG is recruited in social inference, showing increased activity when observers predict a character’s future affect from false beliefs—that is, when an agent’s internal model conflicts with external reality—relative to true-belief judgments (Hooker et al., 2008). Against this background, the enhanced right posterior MTG engagement for tragic relative to joyful themes in our data is plausibly interpreted as rapid socio-semantic construction under representational conflict—characteristic of tragedy’s tension between human will and fate/destiny.

### 4.2 Activity in right LFP

We observed that the right LFP exhibited greater activation in response to tragic than to joyful musical themes. The right LFP is part of Brodmann Area 10 (BA10), which represents one of the most evolutionarily expanded regions in the human brain. BA10 is characterized by pronounced functional segregation: its lateral portion—sometimes categorized as part of the VLPFC—has been associated with working memory, episodic memory retrieval, and emotion regulation, whereas the medial portion is more strongly implicated in self-referential processing and mentalizing tasks (Gilbert et al., 2006; Peng et al., 2018). In the present study, the right LFP may have been co-activated with the left MTG to support episodic memory retrieval and manipulation processes essential for cognitive reappraisal in response to tragic music.

The right LFP has been consistently implicated in the affective evaluation and cognitive regulation of pain. For example, Takahashi et al. (2011) found that muscle pain, compared to cutaneous pain, elicited stronger activation in emotion-related brain regions, particularly the right LFP, suggesting its role in constructing meaning from diffuse and unpleasant sensations and in supporting cognitive reappraisal. Notably, Petrovic et al. (2010) reported greater right LFP and lateral orbitofrontal cortex activity during placebo-induced analgesia compared to opioid-induced analgesia. This finding was interpreted as reflecting enhanced top-down modulation under placebo conditions, wherein the mismatch between a high expectancy of analgesia and ongoing nociceptive input generates prediction error signals that engage prefrontal control mechanisms to attenuate pain. Extending this view, Peng et al. (2018) reviewed evidence that the right LFP is activated in the majority of pain-related paradigms and plays a critical role in the appraisal, affective modulation, and memory-based reconstruction of pain experiences. Rather than functioning solely as a general-purpose cognitive area, the right LFP appears central to generating meaning from aversive sensations and regulating their emotional impact. In light of these findings, the right LFP may serve a comparable function in response to tragic music by facilitating the reappraisal of emotionally negative content. However, we did not obtain in-scanner ratings of reappraisal success. Consequently, our proposal that the right LFP supports the reappraisal of tragic musical content must be regarded as tentative.

Beyond pain regulation, the right LFP is also critically involved in controlling other forms of negative affect. Krendl et al. (2012) demonstrated that regulating emotional responses to stigmatized social targets elicited early activation in prefrontal regions, including the right LFP, particularly during the initial two seconds of downregulation. This suggests the right LFP’s role in rapid, semantically mediated emotional control. Complementarily, Bramson et al. (2020) employed a social–emotional approach–avoidance task, revealing that right LFP activation increased during incongruent conditions (e.g., approaching angry faces or avoiding happy ones), which required participants to override automatic affective action tendencies. These findings converge on the view that the right LFP supports top-down modulation of emotionally aversive or conflicting stimuli. However, the specific nature of this modulation remains ambiguous. The right LFP may subserve either (1) distancing, by suppressing or attenuating negative affect, or (2) embracing, by facilitating reappraisal and semantic reinterpretation. While this functional duality aligns with the Distancing–Embracing model (Menninghaus et al., 2017), it is important to note that the model’s original formulation emphasizes contextual distancing—the pre-emptive recognition of a stimulus as fictional or aesthetic, which provides a protective frame for engaging with negative emotions. In contrast, the right LFP activation observed in our study may reflect a more reactive, emotion-regulatory form of distancing, triggered by the overwhelming emotional salience of the recapitulated tragic theme. This form of distancing suggests a dynamic mechanism whereby cognitive control is recruited to manage intense aesthetic emotions in real time.

### 4.3 Implications

This study offers several implications for both the potential clinical applications of aesthetic tragedy and its theoretical understanding. The meaning-making and cognitive control processes supported by the right LFP may contribute to greater emotional resilience in the face of adverse experiences. Kaldewaij et al. (2021) found that individuals with higher baseline activity in BA10 during emotion regulation exhibited fewer symptoms of post-traumatic stress disorder, suggesting that anterior prefrontal engagement is a neural predictor of resilience. Complementing this, Manassero et al. (2024) showed that repetitive transcranial magnetic stimulation targeting BA10 reduced implicit threat responses and prevented the return of fear, with effects persisting for at least a week. These findings underscore the role of BA10 in modulating negative affect and enhancing long-term emotional regulation. Given the neuroscientific findings in our study, engagement with tragic music has the potential to enhance resilience, insofar as it recruits LFP-supported meaning-making and cognitive control mechanisms that scaffold regulation of negative affect.

This study advances accounts of aesthetic tragedy by showing that vigorous minor-mode music can convey a tragic affect and elicit “internal emotional turmoil or uncertainty.” Our interpretation accords with Carraturo et al. (2025), who report a robust association between the minor mode and negative emotion and note that minor-mode music is less prevalent than major, a relative rarity that may contribute to its negative valence via lower familiarity. We extend this cognitive–affective link by proposing that a principal driver is the minor mode’s greater tonal uncertainty. Relative to major, minor practice more often introduces variable scale degrees and out-of-scale pitches through alterations and mode mixture, destabilizing the tonal frame. As Parncutt (2014) argued, such instability can violate implicit expectations and heighten a general sense of uncertainty, which in turn supports the expression of negative emotion. Within an aesthetic-tragedy framework, this cognitive conflict naturally maps onto the conflict at the heart of tragic narrative.

This conflictual atmosphere can be so strong that even a major-mode sonority acquires negative connotations within a minor-key context. A quintessential example can be found in a participant’s response to the excerpt from the first movement of Brahms’s Piano Concerto No. 1. After the theme’s powerful return in the established key of D minor, the music unexpectedly shifts to an E-major triad—a chromatic chord in D minor. This chord introduces G♯ and B, tones foreign to the D minor scale, creating a moment of sharp tonal contrast that violates the established harmonic context. The participant described this progression as invoking the image of “a powerful villain’s cold, disdainful glare, causing unease.” This response vividly combines a sense of external oppression with the resulting “internal emotional turmoil or uncertainty.” Crucially, this example demonstrates how a major triad, when placed within a threatening minor-key context, can convey a sense of malevolent force. It also underscores the methodological point emphasized by Carraturo et al. (2025): a major weakness in the literature is the failure to distinguish between isolated sounds (e.g., a single chord) and sound progressions governed by tonal-syntactic rules (e.g., a musical passage). Ignoring this contextual difference can yield musically misleading and scientifically inaccurate conclusions. Our argument targets tonal framing rather than chord quality *per se*: affective appraisal is shaped by the scale context and harmonic function that structure expectations. Thus, a major triad can appear threatening within a minor-key context when it functions as a chromatic sonority (e.g., E major in D minor), whereas in isolation—or within a stable major-key frame—major chords are typically appraised as more “happy/safe.” In short, contextual instability and expectancy violation, not the major–minor chord label alone, drive the negative affect.

An expanded view of musical context also includes the influence of elements beyond mode—such as tempo, rhythm, and timbre—on the music’s negative emotional connotations. Whereas Carraturo et al. (2025) treat these elements primarily as potential confounds to be controlled, our ecologically valid stimuli deliberately retain them. In our excerpts, a vigorous tempo, powerful dynamics, strong rhythmic drive, and a dark, weighty timbral profile can, independently or in combination, contribute to the menacing character of minor-mode music. It is therefore unsurprising that our core effects in the left MTG and right LFP diverge from the brain regions highlighted by their meta-analysis. More broadly, the negative affect evoked by minor-mode music extends beyond sadness and likely arises from the interplay of mode, syntactic surprise, texture, melody, dynamics, and timbre.

### 4.4 Limitations

Several limitations of this study should be acknowledged. First, the sample size was relatively small, with a predominance of female participants, and the number of musical excerpts was limited. These factors may have constrained the generalizability and statistical power of the findings. Although future studies should ideally recruit larger and more demographically balanced samples—particularly with respect to gender—and include a broader range of musical expertise, such efforts face increasing challenges. In particular, it is becoming increasingly difficult to recruit participants with sustained interest and familiarity in Western classical music, even within conservatories and music departments. Nonetheless, future work should continue exploring individual differences, including gender and musical training, to better understand how they modulate the neural mechanisms underlying aesthetic engagement with tragic music.

Second, participants’ narrative reports were collected weeks before scanning, we could not confirm that the imagery and affect they described matched their in-scanner experience. Future work should therefore gather trial-by-trial self-ratings of reappraisal effort, emotional intensity, and valence inside the scanner, include quantitative measures of narrative complexity (e.g., linguistic entropy, syntactic depth) as covariates in brain–behavior models, and use connectivity techniques to test whether right LFP activity during tragic passages is functionally coupled with classic cognitive-control hubs. These refinements would directly link subjective regulation success to neural dynamics and clarify whether right LFP recruitment reflects appraisal-based emotion regulation.

Third, although included as a low-tonality comparator, the atonal condition is not affectively neutral and may itself induce negativity or tension. Accordingly, atonal excerpts should be interpreted as controls for tonal structure rather than neutral baselines. Our primary claims therefore hinge on the within-tonal comparison (Tragic > Joyful), which is less sensitive to any valence biases in the atonal material. Nevertheless, the absence of a genuinely neutral auditory control limits the specificity with which we can attribute effects solely to tragic affect. Future work should incorporate affect-neutral baselines to further isolate mode- and narrative-related processes.

Fourth, the relatively low temporal resolution of fMRI limits the ability to precisely dissociate closely occurring musical events. In particular, neural responses associated with participants’ anticipation during the concluding portion of the development section may overlap with the BOLD responses corresponding to the theme recapitulation. Future research could address this limitation by employing methods with higher temporal resolution, such as electroencephalography or magnetoencephalography.

## 5 Conclusion

This study provides novel evidence that the recapitulation of tragic musical themes rapidly engages brain regions associated with semantic integration and high-level cognitive control. These findings illuminate the neurocognitive mechanisms through which listeners actively reframe aesthetically negative experiences, offering empirical support for the Distancing–Embracing model (Menninghaus et al., 2017). Future research should examine how the brain processes distinct types of negatively valenced aesthetic experiences, ranging from the confrontation with tragic fate in heroic narratives to the subdued melancholy and dejection associated with portrayals of everyday life.

## Data Availability

The dataset supporting the findings of this study is publicly available on Mendeley Data at the following https://data.mendeley.com/datasets/g98r5t9wym/1.
